# Multi-trait modeling and machine learning discover new markers associated with stem traits in alfalfa

**DOI:** 10.3389/fpls.2024.1429976

**Published:** 2024-09-09

**Authors:** Cesar A. Medina, Deborah J. Heuschele, Dongyan Zhao, Meng Lin, Craig T. Beil, Moira J. Sheehan, Zhanyou Xu

**Affiliations:** ^1^ Department of Agronomy and Plant Genetics, University of Minnesota, Saint Paul, MN, United States; ^2^ Plant Science Research Unit, USDA-ARS, Saint Paul, MN, United States; ^3^ Breeding Insight, Cornell University, Ithaca, NY, United States

**Keywords:** alfalfa, stem traits, GWAS, multivariate modeling, machine learning

## Abstract

Alfalfa biomass can be fractionated into leaf and stem components. Leaves comprise a protein-rich and highly digestible portion of biomass for ruminant animals, while stems constitute a high fiber and less digestible fraction, representing 50 to 70% of the biomass. However, little attention has focused on stem-related traits, which are a key aspect in improving the nutritional value and intake potential of alfalfa. This study aimed to identify molecular markers associated with four morphological traits in a panel of five populations of alfalfa generated over two cycles of divergent selection based on 16-h and 96-h *in vitro* neutral detergent fiber digestibility in stems. Phenotypic traits of stem color, presence of stem pith cells, winter standability, and winter injury were modeled using univariate and multivariate spatial mixed linear models (MLM), and the predicted values were used as response variables in genome-wide association studies (GWAS). The alfalfa panel was genotyped using a 3K DArTag SNP markers for the evaluation of the genetic structure and GWAS. Principal component and population structure analyses revealed differentiations between populations selected for high- and low-digestibility. Thirteen molecular markers were significantly associated with stem traits using either univariate or multivariate MLM. Additionally, support vector machine (SVM) and random forest (RF) algorithms were implemented to determine marker importance scores for stem traits and validate the GWAS results. The top-ranked markers from SVM and RF aligned with GWAS findings for solid stem pith, winter standability, and winter injury. Additionally, SVM identified additional markers with high variable importance for solid stem pith and winter injury. Most molecular markers were located in coding regions. These markers can facilitate marker-assisted selection to expedite breeding programs to increase winter hardiness or stem palatability.

## 1 Introduction

Alfalfa (*Medicago sativa* L.) is third most valuable field crop in the United States after corn and soybean, with an estimated of 47 million acres harvested in 2022 (USDA National Agricultural Statistics Service, 2022). It is grown for hay, silage, and pasture due to its high nutritive value for milk and muscle mass production in livestock (Higginbotham and Stull, 2009). During harvest, all aerial parts of the plant are collected. Alfalfa biomass yield can be fractionated into leaf and stem components. The leaves comprise a protein-rich, highly digestible portion for ruminant animals, while the stem component represents 50 to 70% of the biomass and comprises a high-fiber, less digestible fraction (Wilman and Altimimi, 1984). Therefore, improving stem digestibility will increase palatability, dry matter, and available energy of the plant especially at later maturity stages.

There are different approaches to increase forage digestibility, including gene knockdowns in the lignin biosynthetic pathway (Guo et al., 2001; Reddy et al., 2005), increasing the proportion of non-lignified tissues (Jung and Lamb, 2006), increasing the leaf/stem ratio (Luckett and Klopfenstein, 1970), or reducing lignin concentration to increase fiber digestibility using traditional breeding (Jung et al., 1997). Previously, a panel of five populations was developed to enhance stem digestibility using recurrent selection for *in vitro* neutral detergent fiber digestibility (IVNDFD) in alfalfa stems (Jung and Lamb, 2006; Xu et al., 2023). Jung and Lamb (2006) generated a cycle zero population (UMN3097) by mixing seeds from six commercial alfalfa varieties. Genotypes of UMN3097 were categorize into four groups: low rapid (16-h) IVNDFD, high rapid (16-h) IVNDFD, low potential (96- h) IVNDFD, and high potential (96-h) IVNDFD. Two cycle 1 divergent stem IVNDFD populations were developed through intercrossing plants with high 16-h and 96-h digestibility (H×H) (UMN3355) and low 16-h and low 96-h digestibility (L×L) (UMN3358). Two cycle 2 divergent stem IVNDFD populations were generated by intermating H×H genotypes of UMN3355 and L×L genotypes of UMN3358 (UMN4016 and UMN4019, respectively). This approach aimed to increase biomass yields while maintaining forage quality and enhancing seasonal stability in stem digestibility.

The stem structure of alfalfa changes during development from young to mature tissues. The young stem has a square shape in cross-section, with major vascular bundles located in the angles, while the mature stem tissues are rounded due to cambial growth (Engels and Jung, 1998). The center of the stem is occupied by the pith, composed of large, compactly arranged parenchyma cells. The parenchyma cells in the pith are unlignified and as stems mature these cells may die, leading to the formation of air-filled cavities in the stem (Teuber and Brick, 1988). The generation of a hollow stem is related to an increased stem lodging and stalk-rot in maize (Colbert I et al., 1987). In alfalfa, stem pith parenchyma is an important trait related to stem maturity, stem water and nutrient content, and palatability (Teuber and Brick, 1988).

Modifications of tissue composition can affect other traits like plant size or susceptibility to diseases (Gallego-Giraldo et al., 2011). Therefore, it is necessary to evaluate different traits that could affect the performance of the selected populations. In the Northern Great Plains of the United States, the ability to develop and maintain an adequate level of freezing resistance is imperative to withstand stressful mid-winter temperatures. During the fall, alfalfa increases tolerance to low temperatures and prepares to enable roots and crowns to survive temperatures as low as -20°C. Winter injury occurs due to cold temperatures in winter that cause freezing of root cells or crown buds, resulting in subsequent damage due to freezing. Sublethal winter injury can decrease vigor during the subsequent growing season (McKenzie et al., 1988).

The availability of the Diversity Arrays Technology (DArT) genotyping panel for alfalfa enables the acquisition of highly consistent 3,000 SNPs to implement genome-wide association studies (GWAS) or genomic selection (Zhao et al., 2023). GWAS can identify marker-trait associations using mixed linear models (MLM) that includes population structure and a kinship matrix to correct false associations (Kang et al., 2008). However, GWAS only analyzes the linear relations for each SNP individually and cannot detect SNP-SNP interactions or small-effect variants. On the other hand, machine learning (ML) models have fewer assumptions about the normality of data and can capture non-linear interactions between the predictor variables (i.e. SNPs) and the response variable. Additionally, ML can capture the combined minor effects of multiple genetic markers, allowing for the development of multi-locus methodologies that consider all SNPs in the model to estimate the importance scores of SNPs. Support vector machines (SVM) and random forest (RF) are two of the most effective machine learning models for predictive analytics capable of improving GWAS results (Roshan et al., 2011; Mieth et al., 2016).

The objective of this study was to identify molecular markers associated with four morphological traits (stem color, presence of stem pith parenchyma [hereafter referred to as stem fill], winter standability, and winter injury) in a panel of five populations of alfalfa generated over two cycles of divergent selection for 16-h and 96-h IVNDFD. In this work, we compared the genotypic response of univariate and multivariate spatial MLM and the markers associated with GWAS and ML models.

## 2 Materials and methods

### 2.1 Plant materials and field experiment

Plant materials were previously detailed by Xu et al. (2023). In summary, a parental population (UMN3097) was created by mixing seeds from six commercial alfalfa varieties (5312, Rushmore, Magnagraze, Wintergreen, Windstar, and WL 325HQ). In the selection cycle zero (C0), approximately 2,400 seeds were hand-sown to establish a plant nursery, and the biomass yield from genotypes was harvested at approximately 25% bloom stage. Fresh biomass yield was subsequently dried at 60°C, and stems and leaves were separated. Each ground stem sample underwent scanning via near-infrared spectroscopy to evaluate 16-h and 96-h *in vitro* neutral detergent fiber digestibility (IVNDFD). The mean values of 16-h and 96-h IVNDFD were utilized to categorize the UMN3097 population into four groups: 117 plants with high 16-h and 96-h digestibility (H16×H96); 33 plants with low 16-h and low 96-h digestibility (L16×L96); 28 plants with high 16-h and low 96-h digestibility (H16×L96); and 26 plants with low 16-h and high 96-h digestibility (L16×H96).

Plants from C0 were intercrossed by hand tripping flowers without emasculation to generate two populations in selection cycle 1 (C1). Populations UMN3355 and UMN3358 were developed through random intercrossing of the plants H16×H96 and L16×L96, respectively. Seeds from C1 populations were established in a spaced plant nursery, following the procedure outlined for the parental population. Biomass yield was harvested, dried, and separated into leaf and stem fractions, and the stem fraction underwent analysis via near-infrared spectroscopy for 16-h and 96-h IVNDFD. Each of the two C1 populations were categorized into four groups using similar criteria applied in the C0 population.

Values of 16-h and 96-h IVNDFD calculated for the C1 populations were used to develop cycle 2 (C2) populations. UMN4016 was generated by intermating approximately 30 plants of UMN3355 with high 16-h and high 96-h IVNDFD, while UMN4019 was generated by intermating approximately 30 plants of UMN3358 with low 16-h and low 96-h IVNDFD (**Supplementary Table 1**). One hundred genotypes from each population were established in 2021 at the University of Minnesota Experimental Research Station in Saint Paul, MN. From the original plant, 12 vegetative cuttings were made with three cuttings planted in each replication. The experimental design used was a randomized complete block design with 1,500 plots arranged in three replicates, with 50 rows and 30 columns. To prevent border effects, a border of the alfalfa cultivar Agate was planted around each side of the plots. The plots were managed following best practices, and pesticides were applied as needed (Xu et al., 2023).

### 2.2 Phenotype collection

A set of four stem traits were collected during 2022 and 2023. Stem color and winter standability were collected on 04/04/22, stem fill was collected on 07/01/22, and winter injury was collected on 04/24/23. All phenotypic values were collected as categorical traits. Stem color and stem fill had two levels: yellow (0) or brown (1) for stem color, and hollow (0) or solid (1) for stem fill. Winter standability was assessed using a modified standability scale ranging from 1 to 5, where 1 represented 0 to 10% erect stems, 2 represented 11 to 30% erect stems, 3 represented 31 to 50% erect stems, 4 represented 51 to 70% erect stems, and 5 represented over 70% erect stems (Johnson et al., 1991). Winter injury was evaluated on a scale from 1 to 5, where:1 indicated dead plants, 2 indicated less than 15% winter injury, and short plants, 3 indicated less than 10% winter injury, short plants with more branches, 4 indicated less than 5% winter injury, tall plants with many branches, 5 indicated no winter injury, tallest plants with many branches. The injury rate was estimated visually without counting the plants.

### 2.3 Univariate and multivariate spatial modeling

The phenotypic response of four stem traits were modeled separately by single trait (ST, i.e. univariate) modeling and conjointly by multi trait (MT, i.e. multivariate) modeling. Each phenotypic response consisted of 1,500 observations corresponding to the plots indexed by 50 rows and 30 columns in a contiguous field array, with three complete blocks and 500 genotypes. Four traits were modeled separately by a univariate mixed model y=Xβ+Zu+ϵ , where **y** is the response variable, **X** and **Z** were incidence matrices for fixed and random effects respectively, β was the vector of fixed effects associated to genotypes, **u** was the vector of random effects associated to blocks, rows and columns, and ϵ was the vector of residuals. The two random components of this model ( u,ϵ ), are assumed to be independent and identically distributed and follow a Normal distribution such that $u\sim N\left(0,\sigma_{g}^{2}I\right)$ and $\epsilon \sim N\left(0,\sigma_{e}^{2}R\right)$ , where I is an identity matrix and R is a variance matrix of vectors of residual variance ( $\sigma_{e}^{2}$ ) for the plot errors by a separable first-order autoregressive model using the field row and column positions (Gilmour et al., 1997). The nugget effect was included to measure the variance to-error variance ratio.

Multivariate spatial modeling uses the same parameters described in the univariate modeling, but data were ordered by traits (*t*) within units in a variance matrix $I_{n}⊗\Sigma$ , where $\Sigma^{\left(t\times t\right)}$ was a factor analytic variance matrix. The error structure was specified with independent units and an unstructured variance matrix.

The ASReml-R software was utilized to estimate the variance components and the best linear unbiased estimates (BLUEs) of the genotypes. BLUEs were used as response variables in genome-wide association studies (GWAS) (Butler et al., 2023). The genetic term was changed as random effect in the same ASReml-R spatial model to obtain the broad sense heritability calculated using the Cullis method (Cullis et al., 2006).


(1) $$H_{Cullis}^{2}=1-\frac{v_{\text{Δ}}^{-BLUP}}{2\sigma_{g}^{2}}\;$$

where $\sigma_{g}^{2}$ is the genetic variance and $v_{\text{Δ}}^{-BLUP}$ is the average standard error of the genotypic best linear unbiased prediction (BLUP).

### 2.4 DArTag genotyping

Genomic representations of 1,502 genotypes were generated by collecting two to three leaflets (~100 mg) per genotype for DNA extraction and genotyping. Leaf tissues were sent to Intertek (Intertek; Alnarp, Sweden) for DNA extraction. The DNA samples were then sent to Diversity Arrays Technology Ltd. (DArT; Canberra, Australia) for genotyping using the 3K DArTag genotyping panel developed by Breeding Insight (Breeding Insight; Ithaca, NY, USA), Cornell University (Zhao et al., 2023).

The alfalfa DArTag panel consists of DNA oligo primers targeting genomic regions containing 3,000 SNP loci distributed across the alfalfa genome (Zhao et al., 2023). DArTag probes hybridize to genomic DNA to capture the target SNP into the DArTag molecule. These DArTag molecules, along with unique barcodes for downstream demultiplexing, are then amplified. Subsequently, DArTag molecules with barcodes are purified and quantified before being sequenced on an Illumina platform (~ 100× per marker per sample). DArT processed the sequencing reads and provided the Allele Match Count Collapsed (AMCC) file containing the read depths of targeted SNPs in the alfalfa SNP array. The AMCC file was then converted into a RADdata object using the `readDArTag` function to export discrete genotypes in a molecular matrix containing the allele dosage with the polyRAD R package v.2.0.0 (Clark et al., 2019). Markers were filtered based on minor allele frequency (MAF) > 0.05, and the number of redundant markers with identical genotype calls were reduced using the `snp.pruning` function of the ASRgenomics v1.1.4 R package (Gezan, 2022). This process resulted in a genotypic matrix with 2,434 SNPs×1,502 genotypes.

### 2.5 Population structure

Principal Component Analysis (PCA) was conducted using the built-in R function `prcomp` with the setting `scale = TRUE`. For population structure analysis, Structure v.2.3.4 software was utilized (Pritchard et al., 2000). PCA and population structure analysis used the genotypic matrix with 2,434 SNPs×1,502 genotypes. To determine the appropriate number of inferred clusters to model the data, ten independent runs were conducted for each number of subpopulations (K) ranging from 2 to 10. The burn-in length was set to 10,000, and the Markov Chain Monte Carlo (MCMC) length was set to 10,000 as well. To identify the optimal number of clusters (K), the Evanno method was employed, where the result with the largest LnP(D) and the smallest K values is considered optimal (Evanno et al., 2005). The optimal K value and the population structure bar plot at K = 2 were generated using the pophelper R package v.2.3.1 (Francis, 2017). The continuous membership values to the two clusters obtained from Structure were included as covariates in the genome-wide association study (GWAS) and can be downloaded from figshare (https://doi.org/10.6084/m9.figshare.25686405.v1).

To evaluate genetic diversity, observed heterozygosity (H_O_), expected heterozygosity within a population (H_S_), and interpopulation differentiation measured with the Rho parameter were calculated using the software GenoDive v.3.0 (Meirmans, 2020).

### 2.6 Linkage disequilibrium

Linkage disequilibrium (LD) between each pair of SNPs was estimated using squared allele-frequency correlations ( $r^{2}$ ) calculated with the ldsep R package v.2.1.5 (Gerard, 2021). The rate of LD decay was estimated using $r^{2}$ and the distances in base pairs (bp) based on the *M. sativa* cultivar XinJiangDaYe reference genome (Chen et al., 2020) using a nonlinear model (Remington et al., 2001). The expected $r^{2}$ value was $E\left(r^{2}\right)=1/\left(1+C\right)$ , where C=4ad , where a is an estimated regression coefficient and d is the physical distance in bp. Assuming a low level of mutation and finite sample size n , the expectation becomes (Hill and Weir, 1988):


(2) $$E\left(r^{2}\right)=\left[\frac{10+C}{\left(2+C\right)\left(11+C\right)}\right]\left[1+\frac{\left(3+C\right)\left(12+4C+C^{2}\right)}{n\left(2+C\left(11+C\right)\right)}\right]\;$$

To evaluate the consistency of LD, global LD decay and estimated LD values for each population were defined as the distance at $r^{2}\;$ = 0.1.

### 2.7 Genome-wide association study and functional annotation

GWAS was conducted using GWASpoly v2.13 (Rosyara et al., 2016) using the Q+K mixed linear model, which conducts single locus analysis incorporating structure information (Q) and a kinship matrix (K) in the model to reduce false positives resulting from population structure and family relatedness. The cluster values obtained from Structure were used as Q, while K was calculated by GWASpoly. K matrix was calculated with the GWASpoly function `set.K` using the leave-one-chromosome-out method, in which a different covariance matrix is calculated for each chromosome based on the markers from all other chromosomes (Yang et al., 2014). The -log_10_(p-values) were corrected by Bonferroni method at a cutoff value of 5% to identify SNPs significantly associated with the traits. Subsequently, significantly associated markers were annotated against the UniProt100 database (Bateman, 2019) using the reference transcriptome dataset for *M. sativa* (Medina et al., 2021) in a window of 84 kb according to LD results.

### 2.8 Machine learning models

Support vector machine (SVM) and random forest (RF) models were implemented to identify linear and non-linear marker-trait associations by fitting all markers simultaneously. SVM find the best hyperplane with the maximal margin in an *p*−dimensional space with respect to a given response variable (Cortes and Vapnik, 1995). A hyperplane refers to a straight line in a high-dimensional or *p*-dimensional space, such as the genotypic matrix with *p* SNPs, where the response variables are the *n* BLUEs. In SVM, each *n*-dimensional input vector ( $x_{i}$ ) of *p* SNP markers is associated with a *y_i_* phenotypic response where $x_{i}\in {\mathbb{R}}^{p}$ and ${\text{y}}_{i}\in \mathbb{R}$ . The following linear regression is performed *f*(x) = (*w,x*) + *b* , where *w*,*x* is the inner product between vectors $w,x\;\in {\mathbb{R}}^{p}$ , *w* is the slope and *b* is the intercept of the hyperplane to be estimated (Liu et al., 2006).

RF is a method to solve both regression and classification problems based on decision trees (Breiman, 2001). A decision tree is a non-parametric algorithm with a hierarchical, tree structure, which consists of a root node, branches, decision nodes, and terminal nodes. The generation of a decision tree involves recursively partitioning the data from the root node based on the available features. The algorithm selects the best features to split the data at each decision node based on certain criteria, such as information gain or the sum of squares (Ishwaran, 2015). The splitting process continues until there is a minimum number of features in a terminal node. RF combines the predictions of multiple decision trees to reduce overfitting and improve the accuracy of the model.

Mean squared error (MSE) of permutation feature importance was used to estimate the variable importance (VI). VI describes how much a prediction model’s accuracy depends on the information in each variable. Therefore, VI is measured by the decrease in prediction accuracy when a covariate is permuted (Breiman, 2001). The purpose of determining the feature or variable importance is to eliminate irrelevant variables to enhance the generalization performance of a model. However, in genetic association studies, machine learning models allow ranking the variables (SNPs) according to how they affect the model predictions. VI of SNPs was calculated to understand how each SNP contributes to the prediction model in the testing model. SVM and RF models were performed using a ten-fold cross-validation with the caret R package v6.0.94 (Kuhn et al., 2019). The VI was ranked from 0 to 100 according to the ranks of each SNP’s impact on the trait and compared with -log10(p-values) from GWAS. Larger VI values indicate a greater increase in the prediction error (MSE) when the SNP is randomly permuted, compared to the MSE value prior to permutation.

## 3 Results

### 3.1 Genotype information

In this study, we genotyped 1,502 individuals from five divergent stem digestibility populations using a mid-density DArTag panel. The five populations used were the cycle zero population (C0); the cycle 1 population with high16-h and 96-h IVNDFD (C1 H×H); the cycle 1 population with low 16-h and 96-h IVNDFD (C1 L×L); the cycle 2 population with high 16-h and 96-h IVNDFD (C2 H×H); and the cycle 2 population with low 16-h and 96-h IVNDFD (C2 L×L) (see plant materials and field experiment in materials and methods section).

**Figure 1:**
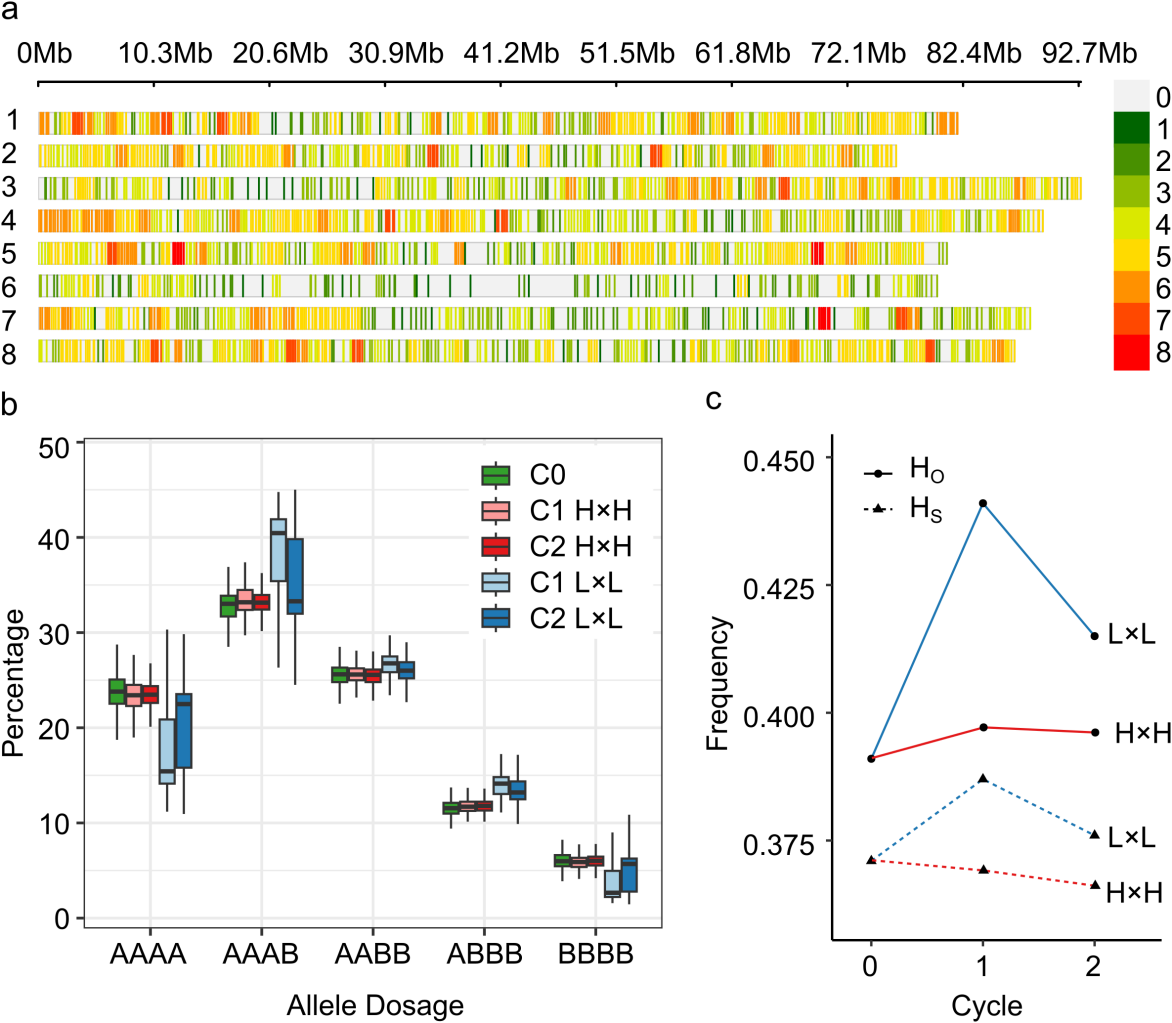
Genotypic information of SNP markers in divergent stem digestibility populations. **(A)** SNP-density plot of 2,434 biallelic SNPs the eight chromosomes of *Medicago sativa*; colors represent number of SNPs within a 1 megabase (Mb) window size. Loci with high-density SNPs are shown in red, and loci with low-density SNPs are shown in green. **(B)** Boxplot of allele dosage in five alfalfa populations. **(A, B)** Represents the reference and alternative allele, respectively. **(C)** Observed (H_O_) and expected heterozygosity (H_S_) by selection cycle. C0, cycle zero population (UMN3097); C1 H×H, cycle 1 population with high 16-h and 96-h vitro neutral detergent fiber digestibility (IVNDFD) (UMN3355); C2 H×H, cycle 2 population with high 16-h and 96-h IVNDFD (UMN4016); C1 L×L, cycle 1 population with low 16-h and 96-h IVNDFD (UMN3358); and C2 L×L, cycle 2 population with low 16-h and 96-h IVNDFD (UMN4019).

Initially, the Allele Match Count Collapsed (AMCC) file contained 3,000 target SNP markers; however, after minor allele frequency (MAF) and collinearity filtering, the genotypic matrix kept 2,434 SNP markers (81%). Markers were not evenly distributed along or among chromosomes (**Figure 1A**). Chromosome 6 had the lowest number of markers (153), with a density of 1.91 SNP/Mb, and a maximum gap between two markers of 4,339 kb around the centromeric region. Chromosome 4 had the highest number of markers (362), with a density of 4.01 SNP/Mb, and a maximum gap between two markers of 1,212 kb. Allele dosage in the five alfalfa populations showed an increase in the heterozygous markers in the C1 and C2 L×L populations (**Figure 1B**). This was corroborated by heterozygosity-based statistics. Observed heterozygosity (H_O_) was greater than the expected heterozygosity within the population (H_S_) in all populations, which is often observed in highly diverse populations. H_O_ was the lowest in the UMN3097 (C0) population (0.391) and highest in the UMN3358 (C1 L×L) population (H_O_ = 0.441). Additionally, the UMN3358 population had the highest value of genetic diversity (H_S_ = 0.387) (**Figure 1C**).

### 3.2 Population structure

**Figure 2:**
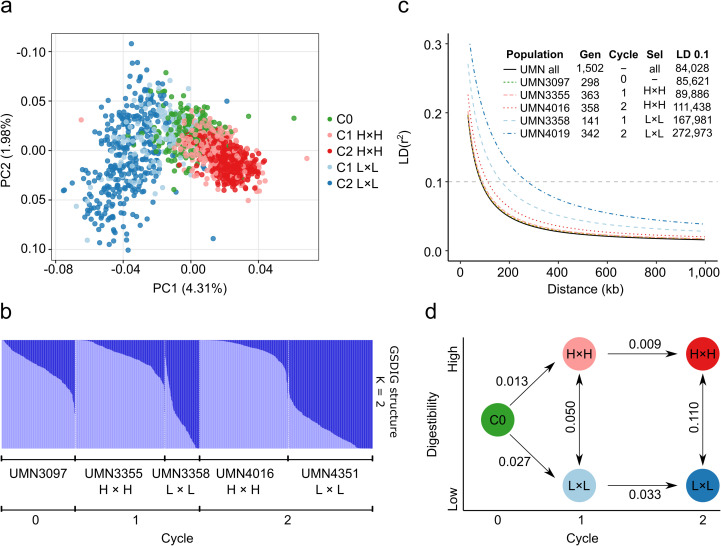
Population structure analysis of divergent stem digestibility populations. **(A)** Principal component analysis for the panel composed of 1,502 genotypes. Colors correspond to the five alfalfa populations. **(B)** Population structure bar plot at K = 2 inferred by the STRUCTURE program for 1,502 genotypes. Each bar represents one genotype, and the bar colors represent the likelihood of membership in each subpopulation. **(C)** Linkage disequilibrium decay of combined (All) populations for five alfalfa populations. The values of half-decay and distances in bp at r^2^ = 0.1 are shown in the inner table. The half values were estimated based on the maximum values of LD decay. Gen = Number of genotypes. Sel = selection criteria. **(D)** Graph of Rho interpopulation distance.

A Principal Component Analysis (PCA) was conducted with 1,502 genotypes to define the population structure. The first and second components contributed 6.29% of the total genetic variance. The individuals of the five populations clustered according to cycle and selection criteria (**Figure 2A**). C0 genotypes were grouped in the middle of the scatter plot (coordinates 0, 0), individuals of cycles 1 and 2 with high digestibility were grouped in the right section of the scatter plot, and individuals of cycles 1 and 2 with low digestibility were grouped in the left section of the scatter plot. C2 populations were more divergent from the base population because of selection. According to Evanno’s ΔK method, the most likely value of K was two, indicating that the 1,502 genotypes could be grouped into two clusters based on differences in their markers (**Supplementary Figure 1**). All genotypes were grouped by populations and ordered according to the membership to the two clusters. Mean values of cluster 1 (dark blue) were significantly different among populations (p-value < 0.01). Mean values of C0 and C1 and C2 high digestibility populations were 0.24, 0.13, and 0.07, respectively, while mean values of low digestibility populations in C1 and C2 were 0.73 and 0.82 (**Figure 2B**).

Linkage disequilibrium (LD) was determined by fitting a non-linear model between the square of the correlation coefficient among pairs of SNPs and physical distances on the *M. sativa* genome. LD decay was measured in all genotypes and in each of the five individual populations. LD decay at r^2^ = 0.1 among all genotypes was the lowest (84 kb), and the values increased with selection cycle. LD decay in the C0 population was the second lowest (86 kb), while C1 and C2 L×L populations had the highest LD decay (168 and 273 kb, respectively) (**Figure 2C**). LD decay was notably higher in L×L populations, doubling and tripling the LD block size in C1 and C2, respectively. Similarly, genetic differentiation values (Rho) were highest between L×L and H×H in C2 (0.11) and lowest between C1 and C2 in H×H populations (0.009). Rho values were doubled when comparing L×L and H×H populations in C1 vs C2, and Rho values were greater in the generation of L×L populations (**Figure 2D**).

### 3.3 Genotypic modeling

**Figure 3:**
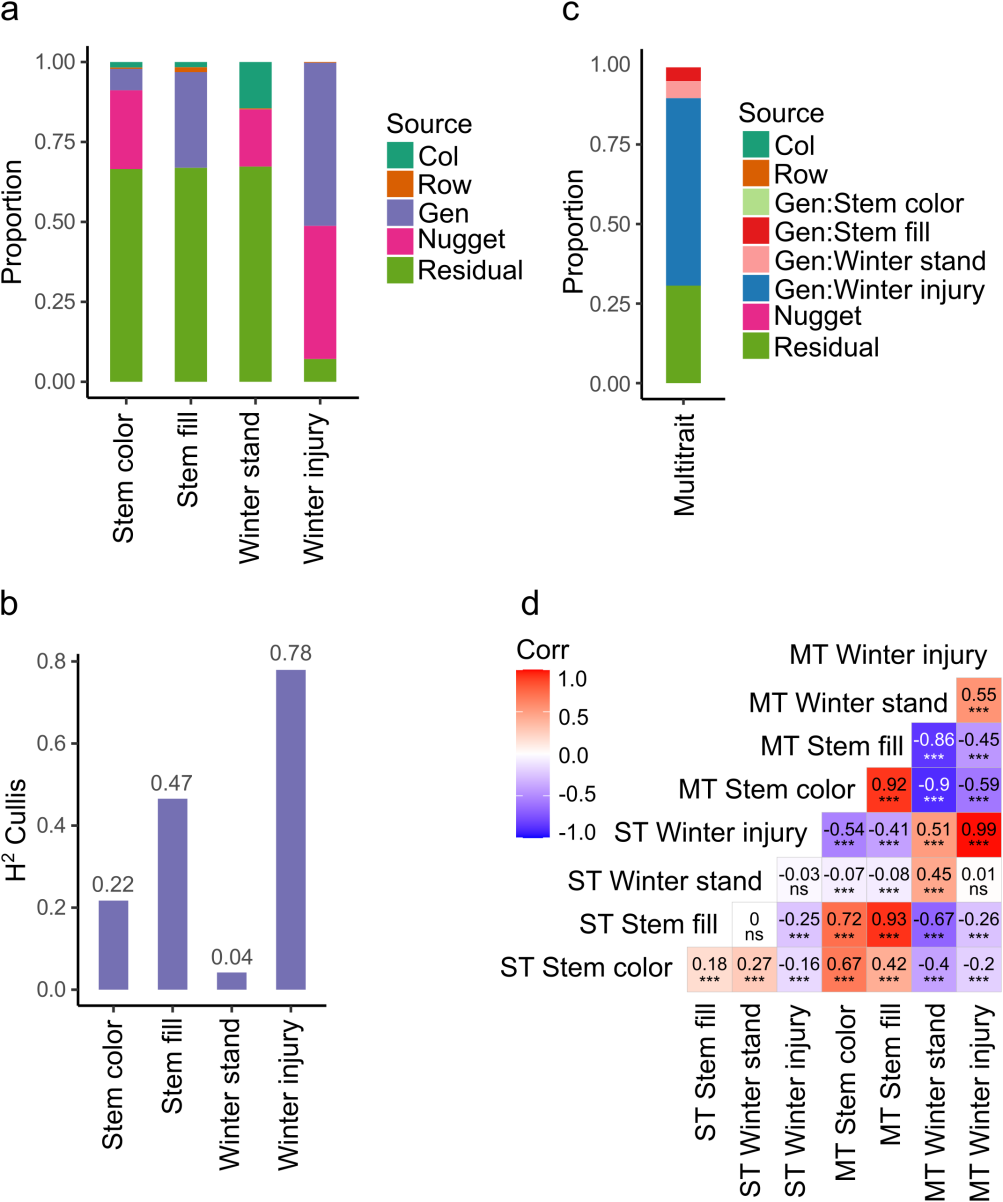
Variance components for random effects in four stem traits. **(A)** Proportion of variance components in four traits modeled using a single trait spatial model. Col and Row are spatial variance effect of columns and rows in the field experiment, Gen is genetic variance, Nugget corresponds to the nugget effect, a measure of error variance in spatial models (Butler et al., 2023). **(B)** Broad sense heritability calculated using the Cullis method (Cullis et al., 2006). **(C)** Proportion of variance components in four traits modeled using multi-trait spatial model. **(D)** Pearson correlation of predicted values of traits associated with stem digestibility by univariate (single trait, ST) and multivariate (multi trait, MT) mixed linear modeling. ***, significant level p-value < 0.0001; ns, non-significative. Winter stand, winter standability.

The best linear unbiased estimates (BLUEs) values were obtained using a single trait (ST) or univariate spatial mixed linear model (MLM). The genotypes and replicates were defined as fixed components, while the nugget effect, columns, and rows were considered random components, and a spatial autoregressive correlation matrix was included as a residual component. Pairwise comparisons among populations showed a significant difference between high and low-digestibility C2 populations in stem color. High and low digestibility C2 populations were significantly different (p-value <0.05) in stem color; and in winter standability (**Supplementary Figure 2**). The Wald test for fixed effects identified that genotypic variation was highly significant (p-value <0.001) for stem color, stem fill, and winter injury, but not for winter standability (**Supplementary Table 2**). The nugget effect was included in the spatial MLM and corresponded to the measure of error variance in the spatial modeling at an infinitesimal separation distance between plots. A log likelihood ratio test showed a significant improvement (p-value <0.001) to the model fit including the nugget effect. Genetic variance was the highest for winter injury (Vg = 0.54) and the lowest for winter standability (Vg = 7.37 ×10^-8^) (**Figure 3A**). The broad sense heritability (H^2^) was high for winter injury (0.78) indicating most of the phenotypic variation of this trait was genetically controlled. H^2^ was medium for stem color (0.22) and stem fill (0.47), and the lowest for winter standability (0.04) (**Figure 3B**).

Multivariate or multi-trait (MT) spatial MLM provided different estimates of genetic variance of the traits by fitting them simultaneously in a model. Winter injury kept the highest genetic variance (Vg = 0.42) while stem color has the lowest genetic variance (Vg = 2.88 ×10^-3^), and winter standability increase this value up to 0.04 (**Figure 3C**). MT-MLM increase the absolute Pearson’s correlation of predicted values between traits. Mean absolute value of Pearson’s correlation in ST-MLM was 0.15 while in MT-MLM was 0.71. BLUEs of winter injury did not change by MT modeling (Pearson’s = 0.99 between ST and MT), but winter standability BLUEs were affected (Pearson’s = 0.45 between ST and MT). The highest Pearson’s correlation in ST modeling was between winter standability and stem color (0.27), and the lowest value was between stem fill and winter injury (-0.25). The highest Pearson’s correlation in MT modeling was between stem fill and stem color (0.92), and the lowest value was between winter standability and stem color (-0.90) (**Figure 3D**).

### 3.4 Genome wide association studies

**Figure 4:**
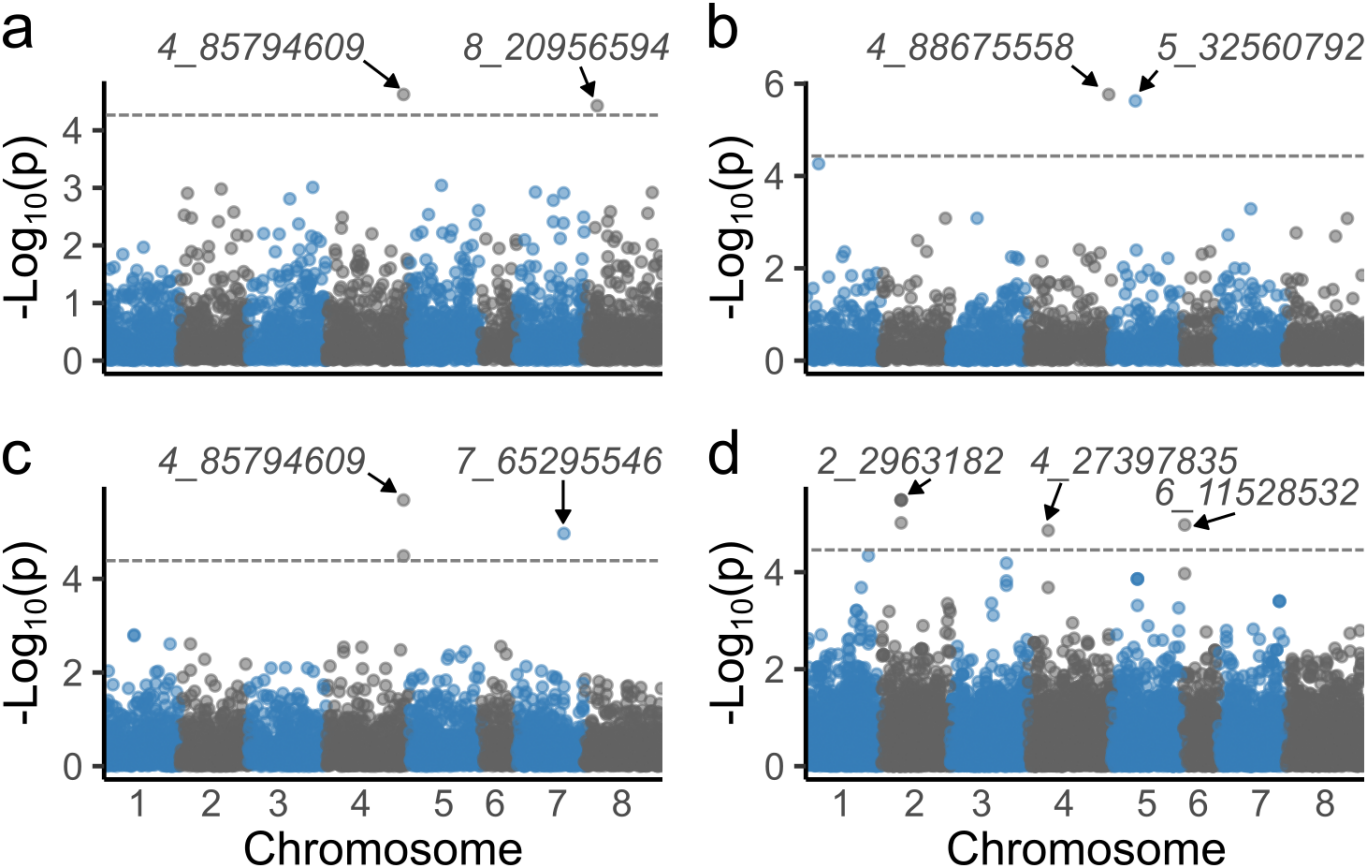
Manhattan plots of significant markers associated with alfalfa stem traits. Significant markers were identified for stem color **(A)**, stem fill **(B)**, winter standability **(C)**, and winter injury **(D)**. The X-axis shows the chromosome numbers, and the Y-axis shows the -log_10_(p-value). The gray line corresponds to the significance threshold (Bonferroni = 0.05).

BLUE values from ST and MT spatial modeling were utilized to identify SNPs associated with stem traits. GWAS with ST-BLUEs identified nine significant associated markers (**Table 1**; **Supplementary Figure 3**). Stem color, stem fill, and winter standability each were associated with two markers, while winter injury was associated with three markers (**Figure 4**). Marker 4_85794609 was associated with two traits: stem color and winter standability. The phenotypic variance explained (R^2^) for each marker shewed minor phenotypic effects. The highest R^2^ was identified in marker 7_65295546, explaining 6% of winter standability (**Table 1**). GWAS with MT-BLUEs identified seven significant associated markers. Stem fill and winter standability each were identified with two markers, winter injury with three markers, and no markers were identified for stem color. The mean -log_10_(p-values) of significant markers was higher in MT-BLUEs (5.25) compared with ST-BLUEs (5.16) (**Table 1**; **Supplementary Figure 3**). Twelve out of thirteen markers were in gene coding regions; however, the distribution of the DArTag markers were sparse across the genome and additional candidate genes were annotated in a window of 84 kb according to LD results (**Supplementary Table 2**).

**Table 1:** List of significant markers and candidate genes for stem traits.

Marker	SNP	ST-BLUEs		MT-BLUEs		Uniprot	Gene	Protein	Ref
		Log	R^2^	Log	R^2^				
Stem color									
4_85794609	A/G	4.62	1.8E-15	2.05	−	UPI000DEDC8CC	*Clu*	Clu domain-containing protein	(Cox and Spradling, 2009)
8_20956594	T/G	4.43	0.005	2.57	−	UPI00078903CC	*RFC2*	Replication factor C subunit 2	(Furukawa et al., 2003)
Stem fill									
3_78077889	G/A	3.77	−	4.56	0.034	−	−	−	
4_88675558	T/C	5.77	0.001	5.50	0.014	A0A2I4GYU2	*FDM1*	Factor of DNA methylation 1-like	(Zheng et al., 2023)
5_32560792	A/G	5.63	3.2E-05	3.26	−	UPI000DEC92ED	*MinE1*	Cell division topological specificity factor	(Maple et al., 2002)
Winter stand.									
3_62442788	T/C	1.16	−	4.7	0.048	A0A445ABY6	*Rix1*	Pre-rRNA-processing protein RIX1	(Castle et al., 2012)
4_85794609	A/G	5.68	0.038	1.4	−	UPI000DEDC8CC	*Clu*	Clu domain-containing protein	(Cox and Spradling, 2009)
7_65295546	C/T	4.97	0.060	1.0	−	UPI00057A69DD	*SEOB*	Sieve element occlusion B	(Ernst et al., 2012)
8_74961533	A/G	0.91	−	5.3	0.025	UPI0010166183	*Rav1*	RAVE complex protein Rav1	(Smardon et al., 2002)
Winter injury									
2_2963182	T/C	5.49	4.2E-04	5.77	0.010	A0A0B2QM34	*PDR1*	Pleiotropic drug resistance protein 1	(Stukkens et al., 2005)
3_74150027	C/T	4.19	−	4.79	0.005	A0A4D6NRG5	*PGL*	6-phosphogluconolactonase	(Xiong et al., 2009)
4_27397835	A/G	4.86	0.002	6.14	0.001	UPI0010168B9C	*WSD1*	O-acyltransferase WSD1-like isoform X2	(Li et al., 2008)
6_11528532	A/G	4.97	0.001	3.44	−	UPI00051B12D6	*KCS*	3-ketoacyl-CoA synthase 20-like	(Kim et al., 2013)

Significant markers were identified using univariate (single trait, ST) and multivariate (multi trait, MT) best linear unbiased estimators (BLUEs). The log is the -log_10_(p-value); R^2^ is the proportion of explained variance. Candidate genes (Gene) were annotated using pan transcriptome information and a monoploid version of *M. sativa* genome (Chen et al., 2020). Winter stand. correspond to winter standability. Gray cells indicate markers below the Bonferroni threshold.

Linkage disequilibrium among markers of interest was tested to identify LD blocks in a window of 2 Mb. Six out of nine significant markers had LD blocks with an average size of 393 kb. Four markers (2_2963182, 4_85794609, 4_88675558, and 8_20956594) were in LD blocks of ~450 kb with other three markers. One marker (4_27397835) was in a block with other two SNPs, and one marker (7_65295546) was in a block with another SNP (**Supplementary Figure 4**). Finally, a list of candidate genes in a window of 84 kb from the marker associated was retrieved identifying interesting genes related with stem traits (**Supplementary Table 3**).

### 3.5 Machine learning predictions

To corroborate the GWAS results, importance scores of markers were calculated using SVM and RF models and compared with GWAS findings. The two markers associated with stem color with ST-BLUEs (4_85794609, 8_20956594) were not associated with MT-BLUEs and had low importance scores (mean of 3.4 and 4.1). Three markers were jointly associated by GWAS using ST and MT-BLUEs in stem fill. Marker 4_88675558 was significantly associated by both ST-BLUEs (5.50) and MT-BLUEs (5.77) and had high importance scores (mean = 81). Marker 3_78077889 was only significantly associated with MT-BLUEs (4.56); however, the mean importance score of SVM and RF was 75.4. Marker 5_32560792 was only significantly associated with ST-BLUEs (5.63), and the mean importance score of SVM and RF was 75.5. Two markers (4_85794609 and 7_65295546) associated with stem fill with MT-BLUEs had the highest importance scores with RF (100 and 99.5). Four markers were significantly associated with winter injury. Markers 2_2963182 and 4_27397835 were significantly associated using both ST-BLUEs (5.49 and 4.86) and MT-BLUEs (5.77 and 6.14) and had high importance scores (mean = 58.9 and 78.9). Two markers (3_74150027, 6_11528532) were significantly associated only with ST-BLUEs or MT-BLUEs, and the mean importance score of SVM and RF was low (mean of 31.1 and 27.2) (**Table 2**).

**Table 2:** Importance scores of significant markers associated with stem traits.

Marker	Trait	ST-BLUEs			MT-BLUES		
		GWAS	SVM	RF	GWAS	SVM	RF
4_85794609	Stem color	4.62	1.5	5.0	2.05	4.0	3.2
8_20956594	Stem color	4.43	1.4	8.0	2.57	5.9	1.1
3_78077889	Stem fill	3.77	100.0	56.8	4.56	100.0	45.0
4_88675558	Stem fill	5.77	59.9	99.8	5.50	64.4	100.0
5_32560792	Stem fill	5.63	78.0	100.0	3.26	80.4	43.6
3_62442788	Winter stand.	1.16	0.8	6.7	4.7	3.0	50.1
4_85794609	Winter stand.	5.68	1.5	100.0	1.4	2.4	13.4
7_65295546	Winter stand.	4.97	82.0	99.5	1.0	0.0	18.5
8_74961533	Winter stand.	0.91	3.2	4.1	5.3	2.8	93.4
2_2963182	Winter injury	5.49	34.9	75.6	5.77	39.9	85.2
3_74150027	Winter injury	4.19	1.9	64.2	4.79	1.8	56.6
4_27397835	Winter injury	4.86	53.6	100.0	6.14	62.0	100.0
6_11528532	Winter injury	4.97	13.1	39.7	3.44	13.2	42.7

Significant markers were identified through genome-wide association studies (GWAS) using univariate (single trait, ST) and multivariate (multi trait, MT) best linear unbiased estimators (BLUEs). GWAS values correspond to -log_10_(p-values). The importance scores for markers identified by GWAS were calculated using support vector machine (SVM) and random forest (RF) models. Importance scores were scaled from 0 to 100. Winter stand. correspond to winter standability. Gray cells indicate markers below the Bonferroni threshold in GWAS or with a variable importance score <10 in SVM or RF models.

Pearson’s correlation of all -log_10_(p-values) from GWAS and all variable importance scores from SVM or RF identified correlated results from multivariate analysis. There are high correlations (>0.8) among importance scores using MT-BLUEs of stem color, stem fill, and winter standability by SVM and GWAS. Winter injury had a high correlation (>0.9) between ST-BLUEs and MT-BLUEs by each model (GWAS, SVM, and RF) but a low correlation among models. For example, the correlation between winter injury SVM and winter injury RF using MT-BLUEs was 0.4 (**Supplementary Figure 5A**). Plotting variable importance of SVM in the genomic position allowed identification of genomic regions with high importance in stem fill and winter injury. In stem fill, four markers with importance scores >90 were located in a region of 6.4 Mb on chromosome 3 (**Supplementary Figure 5B**). For winter injury, seven markers with importance scores >50 were located in a region of 12.4 Mb on chromosome 4 (**Supplementary Figure 5C**).

## 4 Discussion

The alfalfa stem serves as a structural organ supporting all aerial parts and maintaining vascular connections between the roots and leaves. Also, it is an important component of forage yield, representing 50 to 70% of the biomass yield, and influences various traits such as plant height, standability, and digestibility (Wilman and Altimimi, 1984). In this study, we identified 13 molecular markers associated with stem color, stem fill, winter standability, and winter injury in a panel of five divergent stem digestibility populations using a mid-density DArTag panel (Zhao et al., 2023).

### 4.1 Population structure

Previous studies on alfalfa populations and diversity have primarily focused on natural and highly diverse populations from various geographical origins (Qiang et al., 2015; Annicchiarico et al., 2017). In this study, principal component analysis, structure analysis, and interpopulation Rho values revealed clear differentiation between populations with high and low stem digestibility especially during the second selection cycle. Linkage disequilibrium (LD) values ranged from 84 kb to 272 kb, consistent with previous reports in alfalfa (Li et al., 2014). Additionally, LD and Rho values agreed, indicating a greater difference in low digestibility populations (UMN3358 and UMN4019) compared to the C0 population, suggesting a stronger association between alleles in those populations. Low digestibility populations present high heterozygosity compared with cycle zero population. This can be explained because the selected plants used for intermating contained high genetic diversity, altering the genetic diversity of the subsequent populations.

### 4.2 Univariate and multivariate modeling

We modeled the morphological traits using spatial mixed linear models employing both univariate (single trait) and multivariate (multi-trait) models. Univariate modeling serves as the initial step due to its simplicity and reduced computational demand in assessing genetic variance and trait heritability. However, univariate models lack the ability to account for potential relationships between traits since they model each trait independently (Veturi et al., 2012). Therefore, when traits are correlated, multivariate models can provide more accurate predicted values. Multivariate genetic analysis has been applied in genomic selection obtaining higher predicted abilities compared from univariate modeling.

Multivariate models, designed to capture complex trait relationships, often produce more accurate parameter estimates and predictions than univariate models (Isik et al., 2017). In our study, we observed low Pearson correlations among traits using univariate models (-0.25 to 0.27) and higher correlations in multivariate models (-0.9 to 0.92). Predicted values of winter injury and stem fill remained consistent across univariate and multivariate modeling. However, predicted values of traits with low heritability like stem color and winter standability differed between the multivariate and univariate models (Pearson correlation of 0.67 and 0.45, respectively), as they were influenced by observations in other traits. These discrepancies affected the molecular markers associated with GWAS. In this work, nine, seven, and three markers were associated with univariate, multivariate, or both modeling approaches, respectively. Multivariate data analysis identified four new markers that were under the Bonferroni threshold in univariate data analysis. However, six markers identified by univariate data analysis were under the Bonferroni threshold in multivariate analysis. It has been recommended that both multivariate and univariate models could be useful for identifying candidate loci with potential effects for later biological experiments (Fernandes et al., 2021).

### 4.3 Stem fill

Stem fill can be considered a measure of plant maturity and is related to a reduction in winter standability and resistance to stem lodging in maize (Colbert I et al., 1987). In this study, we identified an inverse correlation between winter standability and solid stems (-0.86). One possible explanation is the maturity stage of alfalfa genotypes. In young stems, highly digestible pith parenchyma cells are intact, but there are also lower lignified tissues, decreasing the percentage of erect stems. The unlignified pith parenchyma cells die in mature stems, leading to the formation of a hollow stem, and increasing the lignified tissues and the winter standability.

Three markers were associated with stem fill, two of them located in coding regions. The gene containing the marker 4_88675558 encodes a factor of DNA methylation 1-like (*FDM1*). *FDM1* has been reported to control plant and organ size in woodland strawberry by controlling the expression of multiple genes related to the cell cycle and cytoskeleton organization (Zheng et al., 2023). Marker 5_32560792 was located in a region annotated as a cell division topological specificity factor (*MinE1*). *MinE1* has been reported to control plastid division in *Arabidopsis* (Maple et al., 2002). Finally, marker 3_78077889 was not located in a coding region. However, this is one of the four markers with variable importance > 90 with support vector machine located in a region of 6.4 Mb on chromosome three (**Supplementary Figure 5B**).

### 4.4 Stem color and winter standability

In this study, we identified a negative correlation between winter standability and stem color (-0.9), i.e., brown stem color was associated with low winter standability. This result could be explained by a loss of water pressure in brown stems, resulting in low standability. Freezing injury in alfalfa results from the pressure exerted by intracellular ice crystals, which disrupt the membrane structure during the thawing process. The damage occurs in older parenchyma cells of the phloem and xylem, as well as in the central pith-like structure of the upper part of the taproot (McKenzie et al., 1988). Multivariate analysis showed significant differences (p-value < 0.05) in C2 populations for stem color and winter standability (**Supplementary Figure 2**). C2 L×L (UMN4019) had yellow and erect stems while C2 H×H (UMN4016) were brown and prostrate stems, which suggest that low digestible stems can be more tolerant to effect of cold winter temperatures.

Marker 4_85794609 was associated with stem color and winter standability. This marker explains 4% of variation in winter standability and it was located in a locus coding for a Clu-domain-containing protein. In plant cells, Clu was postulated to control mitochondrial binding and localization by regulating the interaction with microtubules known to assist with cellular structure with water tension (Logan, 2010). Marker 8_20956594, associated with stem color, was located in a gene annotated as replication factor C subunit 2 (*RFC2*). *RFC2* is involved in DNA replication and repair mechanisms, with high expression in proliferating tissues such as the shoot apical meristem and very weakly in non-proliferating tissues (Furukawa et al., 2003). Marker 7_65295546, which explained 6% of the phenotypic variation in winter standability, was located in a gene annotated as sieve element occlusion B (*SEOB*). *SEO* genes encode P-proteins to facilitate rapid wound sealing after injury, preventing the loss of turgor and photosynthate (Ernst et al., 2012). In *Medicago truncatula, MtSEO-F1-F3* are specifically expressed in immature sieve elements (Noll et al., 2009). Additionally, marker 3_62442788 associated with winter standability was located at 37 kb upstream of other *SEOB* gene (**Supplementary Table 3**). We can hypothesize that *RFC2* or *SEOB* genes have roles in responding to plant injury and keeping cell turgor for stem standability after cold winter temperatures.

### 4.5 Winter injury

Winter injury was the trait with highest genetic variance and broad sense heritability (H²) after genetic data analysis. Although stem color and winter standability were measured during a similar season one year before, their genetic variance and the H² were lowest compared with winter injury. The high H² (0.78) of winter injury indicate that most of the phenotypic variations of this trait were genetically controlled and offers more confidence in the results obtained in this work. Stem traits evaluated in this work are not commonly evaluated in breeding programs and the information reported here is an important guideline for forage selection.

Four markers were associated with winter injury, and all of them were in coding regions. Marker 2_2963182 was located in a locus annotated as plasma membrane pleiotropic drug resistance protein 1 (*PDR1*). *PDR1* is an ATP-binding cassette transporter related to plant defense against different fungal and oomycete pathogens (Stukkens et al., 2005; Bultreys et al., 2009). Similarly, marker 3_74150027 was located in the pathogenesis-related gene, 6-phosphogluconolactonase *(PGL*). Mutants *pgl3* plants exhibit enhanced resistance to *Pseudomonas syringae* pv. *maculicola* and *Hyaloperonospora arabidopsidis*, and *PGL3* gene is an essential gene for plant size and development (Xiong et al., 2009). Markers 4_27397835 and 6_11528532 were located in loci annotated as O-acyltransferase WSD1-like isoform X2 (*WSD1*) and 3-ketoacyl-CoA synthase 20-like (*KCS*), respectively. Both genes are involved in the wax biosynthesis pathway (Lewandowska et al., 2020). WSD1 functions as wax ester biosynthesis in stems (Li et al., 2008), and KCS participates in the synthesis of fatty acid elongation and cuticular wax biosynthetic pathways (Kim et al., 2013). Our findings highlight the possible importance of the stem wax biosynthesis pathway in cold tolerance in alfalfa and agree with previous reports. For example, in *Arabidopsis*, the *dewax* mutant showed a greater ability to accumulate waxes under cold acclimation and displayed freezing tolerance at colder temperatures compared with the wild type (Rahman et al., 2021).

### 4.6 Validation of candidate markers by machine learning models

Breeders need stable targeted markers to track the transmission of traits through different breeding cycles. In this work we identified 13 markers associated with stem traits using GWASpoly (Rosyara et al., 2016). Although GWAS is a comprehensive approach to systematically search the genome for causal genetic variation, new tools like machine learning (ML) models can enhance the detection of markers associated with traits of interest.

In ML models, all SNPs are ranked based on their variable importance (VI) on a scale from 0 to 100. Larger values indicate a greater increase in the prediction error (mean squared error, MSE) when the SNP is randomly permuted, compared to the MSE value prior to permutation. ML models can corroborate GWAS results and enhance the detection of markers associated with traits of interest (Roshan et al., 2011). In this study, support vector machine (SVM) and random forest (RF) confirmed the significance of 11 out of 13 markers identified by GWAS. Additionally, SVM identified two regions of 6.4 Mb and 12.4 Mb with four and seven markers with high variable importance for stem fill and winter injury, respectively. Therefore, ML enhanced the ability to detect new genetic associations with various traits, addressing the challenges posed by the complex genetic architecture of quantitative traits. SVM exhibited lower shrinkage of VI compared with RF. However, both methods are valid and commentary to classical GWAS.

In conclusion, this work demonstrates how the use of multivariate mixed linear models and machine learning can broaden the molecular markers associated with four important traits in alfalfa. The DArTag genotyping panel used in this study has three advantages: 1. It demonstrated that assortative mating during two selection cycles changed the population structure and genetic diversity between low and high digestibility populations. 2. Genotypic information was utilized to identify associated markers with four morphological traits using GWAS and ML models. 3. The markers identified in this work highlight important mechanisms controlling stem traits such as stem fill or winter survival. DArTag markers identified in this study are highly reproducible in other populations and can facilitate marker-assisted selection to increase winter hardiness or palatability in alfalfa. A next bidirectional selection cycle is in progress and data of IVNDFD such as other forage quality traits will be included in the next part of this project.

## Data Availability

Large data sets, including genotypic matrix, BLUE values, Structure cluster membership values, and variable importance scores from support vector machine and random forest are available in figshare (https://doi.org/10.6084/m9.figshare.25686405.v1).

## References

[B1] Annicchiarico P. Wei Y. Brummer E. C. (2017). Genetic structure of putative heterotic populations of alfalfa. Plant Breed. 136, 671–678. doi: 10.1111/pbr.12511

[B2] Bateman A. (2019). UniProt: a worldwide hub of protein knowledge. Nucleic Acids Res. 47, D506–D515. doi: 10.1093/nar/gky1049 30395287 PMC6323992

[B3] Breiman L. (2001). Random forests. Mach. Learn 45, 5–32. doi: 10.1023/A:1010933404324

[B4] Bultreys A. Trombik T. Drozak A. Boutry M. (2009). *Nicotiana plumbaginifolia* plants silenced for the ATP-binding cassette transporter gene NpPDR1 show increased susceptibility to a group of fungal and oomycete pathogens. Mol. Plant Pathol. 10, 651–663. doi: 10.1111/j.1364-3703.2009.00562.x 19694955 PMC6640336

[B5] Butler D. G. Cullis B. R. Gilmour A. R. Gogel B. J. Thompson R. Biology C. (2023). ASReml-R reference manual. Available online at: https://vsni.co.uk/. (Accessed August 18, 2024)

[B6] Castle C. D. Cassimere E. K. Denicourt C. (2012). LAS1L interacts with the mammalian Rix1 complex to regulate ribosome biogenesis. Mol. Biol. Cell 23, 716–728. doi: 10.1091/mbc.E11-06-0530 22190735 PMC3279398

[B7] Chen H. Zeng Y. Yang Y. Huang L. Tang B. Zhang H.. (2020). Allele-aware chromosome-level genome assembly and efficient transgene-free genome editing for the autotetraploid cultivated alfalfa. Nat. Commun. 11, 2494. doi: 10.1038/s41467-020-16338-x 32427850 PMC7237683

[B8] Clark L. V. Lipka A. E. Sacks E. J. (2019). polyRAD: genotype calling with uncertainty from sequencing data in polyploids and diploids. G3 Genes Genomes Genet. 9, 663–673. doi: 10.1534/g3.118.200913 PMC6404598 30655271

[B9] Colbert I T. R. Kang M. S. Myers O. Zuber M. S. (1987). General and specific combining ability estimates for pith cell death in stalk internodes of maize. Field Crops Res 17, 155–161. doi: 10.1016/0378-4290(87)90089-X

[B10] Cortes C. Vapnik V. (1995). Support-vector networks. Mach. Learn 20, 273–297. doi: 10.1007/BF00994018

[B11] Cox R. T. Spradling A. C. (2009). clueless, a conserved Drosophila gene required for mitochondrial subcellular localization, interacts genetically with parkin Remove 4-colour Black 499, 490–499. doi: 10.1242/dmm.002378 PMC2737057 19638420

[B12] Cullis B. R. Smith A. B. Coombes N. E. (2006). On the design of early generation variety trials with correlated data. J. Agric. Biol. Environ. Stat. 11, 381–393. doi: 10.1198/108571106X154443

[B13] Engels F. M. Jung H. G. (1998). Alfalfa stem tissues: cell-wall development and lignification. Ann. Bot. 82, 561–568. doi: 10.1006/anbo.1998.0705

[B14] Ernst A. M. Jekat S. B. Zielonka S. Müller B. Neumann U. Rüping B.. (2012). Sieve element occlusion (SEO) genes encode structural phloem proteins involved in wound sealing of the phloem. Proc. Natl. Acad. Sci. U.S.A. 109, E1980–E1989. doi: 10.1073/pnas.1202999109 22733783 PMC3396537

[B15] Evanno G. Regnaut S. Goudet J. (2005). Detecting the number of clusters of individuals using the software structure: a simulation study. Mol. Ecol. 14, 2611–2620. doi: 10.1111/j.1365-294X.2005.02553.x 15969739

[B16] Fernandes S. B. Zhang K. S. Jamann T. M. Lipka A. E. (2021). How well can multivariate and univariate GWAS distinguish between true and spurious pleiotropy? Front. Genet. 11. doi: 10.3389/fgene.2020.602526 PMC7873880 33584799

[B17] Francis R. M. (2017). pophelper: an R package and web app to analyse and visualize population structure. Mol. Ecol. Resour 17, 27–32. doi: 10.1111/1755-0998.12509 26850166

[B18] Furukawa T. Ishibashi T. Kimura S. Tanaka H. Hashimoto J. Sakaguchi K. (2003). Characterization of all the subunits of replication factor C from a higher plant, rice (*Oryza sativa* L.), and their relation to development. Plant Mol. Biol. 53, 15–25. doi: 10.1023/B:PLAN.0000009258.04711.62 14756303

[B19] Gallego-Giraldo L. Jikumaru Y. Kamiya Y. Tang Y. Dixon R. A. (2011). Selective lignin downregulation leads to constitutive defense response expression in alfalfa (*Medicago sativa* L.). New Phytol. 190, 627–639. doi: 10.1111/j.1469-8137.2010.03621.x 21251001

[B20] Gerard D. (2021). Scalable bias-corrected linkage disequilibrium estimation under genotype uncertainty. Heredity (Edinb) 127, 357–362. doi: 10.1038/s41437-021-00462-5 34373594 PMC8479074

[B21] Gezan S. (2022). ASRgenomics. Available online at: https://cran.r-project.org/web/packages/ASRgenomics/ASRgenomics.pdf (Accessed May 6, 2024).

[B22] Gilmour A. R. Cullis B. R. Verbyla A. P. (1997). Accounting for natural and extraneous variation in the analysis of field experiments. J. Agric. Biol. Environ. Stat. 2, 269–293. doi: 10.2307/1400446

[B23] Guo D. Chen F. Wheeler J. Winder J. Selman S. Peterson M.. (2001). Improvement of in-rumen digestibility of alfalfa forage by genetic manipulation of lignin O-methyltransferases. Transgenic Res. 10, 457–464. doi: 10.1023/a:1012278106147 10.1023/a:1012278106147 11708655

[B24] Higginbotham G. E. Stull C. L. (2009).Alfalfa utilization by livestock. In: Irrigated alfalfa management in Mediterranean and Desert zones (Oakland, CA: University of California Agriculture and Natural Resources). Available online at: https://alfalfa.ucdavis.edu/sites/g/files/dgvnsk12586/files/media/documents/UCAlfalfa8303Livestock-reg.pdf (Accessed April 11, 2024).

[B25] Hill W. G. Weir B. S. (1988). Variances and covariances of squared linkage disequilibria in finite populations. Theor. Popul Biol. 33, 54–78. doi: 10.1016/0040-5809(88)90004-4 3376052

[B26] Ishwaran H. (2015). The effect of splitting on random forests. Mach. Learn 99, 75–118. doi: 10.1007/s10994-014-5451-2 PMC5599182 28919667

[B27] Isik F. Holland J. Maltecca C. (2017). “Multivariate models,” in Genetic data analysis for plant and animal breeding. Eds. Isik F. Holland J. Maltecca C. (Springer International Publishing, Cham), 165–201. doi: 10.1007/978-3-319-55177-7_6

[B28] Johnson D. Miller D. Sharpee D. Darling M. Hoard G. Miller D.. (1991). Standability expression (lodging resistance). Available online at: https://www.naaic.org/stdtests/updated/pdfs/StandabilityExpression_updated.pdf (Accessed May 1, 2024).

[B29] Jung H. G. Lamb J. F. S. (2006). Stem morphological and cell wall traits associated with divergent in *vitro* neutral detergent fiber digestibility in alfalfa clones. Crop Sci. 46, 2054–2061. doi: 10.2135/cropsci2005.12.0470

[B30] Jung H. G. Mertens D. R. Payne A. J. (1997). Correlation of acid detergent lignin and Klason lignin with digestibility of forage dry matter and neutral detergent Fiber. J. Dairy Sci. 80, 1622–1628. doi: 10.3168/jds.S0022-0302(97)76093-4 9276801

[B31] Kang H. M. Zaitlen N. A. Wade C. M. Kirby A. Heckerman D. Daly M. J.. (2008). Efficient control of population structure in model organism association mapping. Genetics 178, 1709–1723. doi: 10.1534/genetics.107.080101 18385116 PMC2278096

[B32] Kim J. Jung J. H. Lee S. B. Go Y. S. Kim H. J. Cahoon R.. (2013). Arabidopsis 3-ketoacyl-coenzyme a synthase9 is involved in the synthesis of tetracosanoic acids as precursors of cuticular waxes, suberins, sphingolipids, and phospholipids. Plant Physiol. 162, 567–580. doi: 10.1104/pp.112.210450 23585652 PMC3668053

[B33] Kuhn M. Contributions from Wing J. Weston S. Williams A. Keefer C. Engelhardt A.. (2019). caret: classification and regression training. Available online at: https://cran.r-project.org/package=caret (Accessed March 20, 2020).

[B34] Lewandowska M. Keyl A. Feussner I. (2020). Wax biosynthesis in response to danger: its regulation upon abiotic and biotic stress. New Phytol. 227, 698–713. doi: 10.1111/nph.16571 32242934

[B35] Li F. Wu X. Lam P. Bird D. Zheng H. Samuels L.. (2008). Identification of the wax ester synthase/acyl-coenzyme a:diacylglycerol acyltransferase WSD1 required for stem wax ester biosynthesis in Arabidopsis. Plant Physiol. 148, 97–107. doi: 10.1104/pp.108.123471 18621978 PMC2528131

[B36] Li X. Han Y. Wei Y. Acharya A. Farmer A. D. Ho J.. (2014). Development of an alfalfa SNP array and its use to evaluate patterns of population structure and linkage disequilibrium. PloS One 9, e84329. doi: 10.1371/journal.pone.0084329 24416217 PMC3887001

[B37] Liu W. Meng X. Xu Q. Flower D. R. Li T. (2006). Quantitative prediction of mouse class I MHC peptide binding affinity using support vector machine regression (SVR) models. BMC Bioinf. 7, 182. doi: 10.1186/1471-2105-7-182 PMC1513606 16579851

[B38] Logan D. C. (2010). Mitochondrial fusion, division and positioning in plants. Biochem. Soc. Trans. 38, 789–795. doi: 10.1042/BST0380789 20491666

[B39] Luckett C. R. Klopfenstein T. J. (1970). Leaf-to-stem ratio and composition of alfalfa from five harvesting systems. J. Anim. Sci. 31, 126–129. doi: 10.2527/jas1970.311126x

[B40] Maple J. Chua N. H. Møller Simon G. (2002). The topological specificity factor AtMinE1 is essential for correct plastid division site placement in Arabidopsis. Plant J. 31, 269–277. doi: 10.1046/j.1365-313X.2002.01358.x 12164807

[B41] McKenzie J. S. Paquin R. Duke S. H. (1988). “Cold and heat tolerance,” in Alfalfa and alfalfa improvement, 259–302. doi: 10.2134/agronmonogr29.c8

[B42] Medina C. A. Samac D. A. Yu L.-X. (2021). Pan-transcriptome identifying master genes and regulation network in response to drought and salt stresses in Alfalfa (*Medicago sativa* L.). Sci. Rep. 11, 17203. doi: 10.1038/s41598-021-96712-x 34446782 PMC8390513

[B43] Meirmans P. G. (2020). GENODIVE version 3.0: easy-to-use software for the analysis of genetic data of diploids and polyploids. Mol. Ecol. Resour 20, 1126–1131. doi: 10.1111/1755-0998.13145 32061017 PMC7496249

[B44] Mieth B. Kloft M. Rodríguez J. A. Sonnenburg S. Vobruba R. Morcillo-Suárez C.. (2016). Combining multiple hypothesis testing with machine learning increases the statistical power of genome-wide association studies. Sci. Rep. 6, 1–14. doi: 10.1038/srep36671 PMC5125008 27892471

[B45] Noll G. A. Rüping B. Ernst A. M. Bucsenez M. Twyman R. M. Fischer R.. (2009). The Promoters of forisome genes *MtSEO2* and *MtSEO3* direct gene expression to immature sieve elements in *Medicago truncatula* and *Nicotiana tabacum* . Plant Mol. Biol. Rep. 27, 526–533. doi: 10.1007/s11105-009-0120-5

[B46] Pritchard J. K. Stephens M. Donnelly P. (2000). Inference of population structure using multilocus genotype data. Genetics 155, 945–959. doi: 10.1093/genetics/155.2.945 10835412 PMC1461096

[B47] Qiang H. Chen Z. Zhang Z. Wang X. Gao H. Wang Z. (2015). Molecular diversity and population structure of a worldwide collection of cultivated tetraploid alfalfa (*Medicago sativa* subsp. *sativa* L.) germplasm as revealed by microsatellite markers. PloS One 10, 1–12. doi: 10.1371/journal.pone.0124592 PMC4406709 25901573

[B48] Rahman T. Shao M. Pahari S. Venglat P. Soolanayakanahally R. Qiu X.. (2021). Dissecting the roles of cuticular wax in plant resistance to shoot dehydration and low-temperature stress in Arabidopsis. Int. J. Mol. Sci. 22, 1–21. doi: 10.3390/ijms22041554 PMC7913816 33557073

[B49] Reddy M. S. S. Chen F. Shadle G. Jackson L. Aljoe H. Dixon R. A. (2005). Targeted down-regulation of cytochrome P450 enzymes for forage quality improvement in alfalfa (*Medicago sativa* L.). Proc. Natl. Acad. Sci. 102, 16573–16578. doi: 10.1073/pnas.0505749102 16263933 PMC1283808

[B50] Remington D. L. Thornsberry J. M. Matsuoka Y. Wilson L. M. Whitt S. R. Doebley J.. (2001). Structure of linkage disequilibrium and phenotypic associations in the maize genome. Proc. Natl. Acad. Sci. 98, 11479–11484. doi: 10.1073/pnas.201394398 11562485 PMC58755

[B51] Roshan U. Chikkagoudar S. Wei Z. Wang K. Hakonarson H. (2011). Ranking causal variants and associated regions in genome-wide association studies by the support vector machine and random forest. Nucleic Acids Res. 39, e62–e62. doi: 10.1093/nar/gkr064 21317188 PMC3089490

[B52] Rosyara U. R. De Jong W. S. Douches D. S. Endelman J. B. (2016). Software for genome-wide association studies in autopolyploids and its application to potato. Plant Genome 9, 1–10. doi: 10.3835/plantgenome2015.08.0073 27898814

[B53] Smardon A. M. Tarsio M. Kane P. M. (2002). The RAVE complex is essential for stable assembly of the yeast V-ATpase. J. Biol. Chem. 277, 13831–13839. doi: 10.1074/jbc.M200682200 11844802

[B54] Stukkens Y. Bultreys A. Grec S. Trombik T. Vanham D. Boutry M. (2005). NpPDR1, a pleiotropic drug resistance-type ATP-binding cassette transporter from *nicotiana plumbaginifolia*, plays a major role in plant pathogen defense. Plant Physiol. 139, 341–352. doi: 10.1104/pp.105.062372 16126865 PMC1203383

[B55] Teuber L. R. Brick M. A. (1988). “Morphology and anatomy,” in Alfalfa and alfalfa improvement, 125–162. doi: 10.2134/agronmonogr29.c4

[B56] USDA National Agricultural Statistics Service (2022). U.S. Census of Agriculture. Census of Agriculture 1. Available at: https://www.nass.usda.gov/AgCensus/ (Accessed August 18, 2024).

[B57] Veturi Y. Kump K. Walsh E. Ott O. Poland J. Kolkman J. M.. (2012). Multivariate mixed linear model analysis of longitudinal data: An information-rich statistical technique for analyzing plant disease resistance. Phytopathology 102, 1016–1025. doi: 10.1094/PHYTO-10-11-0268 23046207

[B58] Wilman D. Altimimi M. A. K. (1984). The in-vitro digestibility and chemical composition of plant parts in white clover, red clover and lucerne during primary growth. J. Sci. Food Agric. 35, 133–138. doi: 10.1002/jsfa.2740350203

[B59] Xiong Y. Defraia C. Williams D. Zhang X. Mou Z. (2009). Characterization of Arabidopsis 6-phosphogluconolactonase T-DNA insertion mutants reveals an essential role for the oxidative section of the plastidic pentose phosphate pathway in plant growth and development. Plant Cell Physiol. 50, 1277–1291. doi: 10.1093/pcp/pcp070 19457984

[B60] Xu Z. Heuschele D. J. Lamb J. F. S. Jung H.-J. G. Samac D. A. (2023). Improved Forage Quality in Alfalfa (*Medicago sativa* L.) via Selection for Increased Stem Fiber Digestibility. Agronomy 13, 770. doi: 10.3390/agronomy13030770

[B61] Yang J. Zaitlen N. A. Goddard M. E. Visscher P. M. Price A. L. (2014). Advantages and pitfalls in the application of mixed-model association methods. Nat. Genet. 46, 100–106. doi: 10.1038/ng.2876 PMC3989144 24473328

[B62] Zhao D. Mejia-Guerra K. M. Mollinari M. Samac D. Irish B. Heller-Uszynska K.. (2023). A public mid-density genotyping platform for alfalfa (*Medicago sativa* L.). Genet. Resour. 4, 55–63. doi: 10.46265/genresj.EMOR6509

[B63] Zheng G. Hu S. Cheng S. Wang L. Kan L. Wang Z.. (2023). Factor of DNA methylation 1 affects woodland strawberry plant stature and organ size via DNA methylation. Plant Physiol. 191, 335–351. doi: 10.1093/plphys/kiac462 36200851 PMC9806633

