# Supplementary material for: Multi-trait modeling and machine learning discover new markers associated with stem traits in alfalfa
Source: Front Plant Sci. 2024 Sep 9;15:1429976. doi: 10.3389/fpls.2024.1429976 (PMC11418689; doi:10.3389/fpls.2024.1429976)
Supplement: Supplementary file 1 [file DataSheet1.docx]

Supplementary Material

# Supplementary Figures


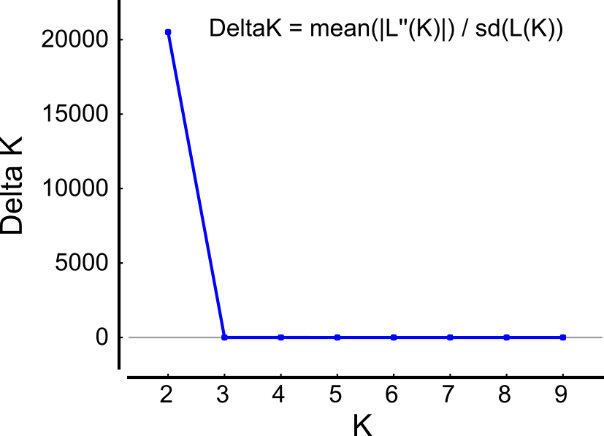


**Supplementary Figure 1. Estimation of the number of subpopulations using the STRUCTURE program.** Delta K = 2 is the most likely number of subpopulations.


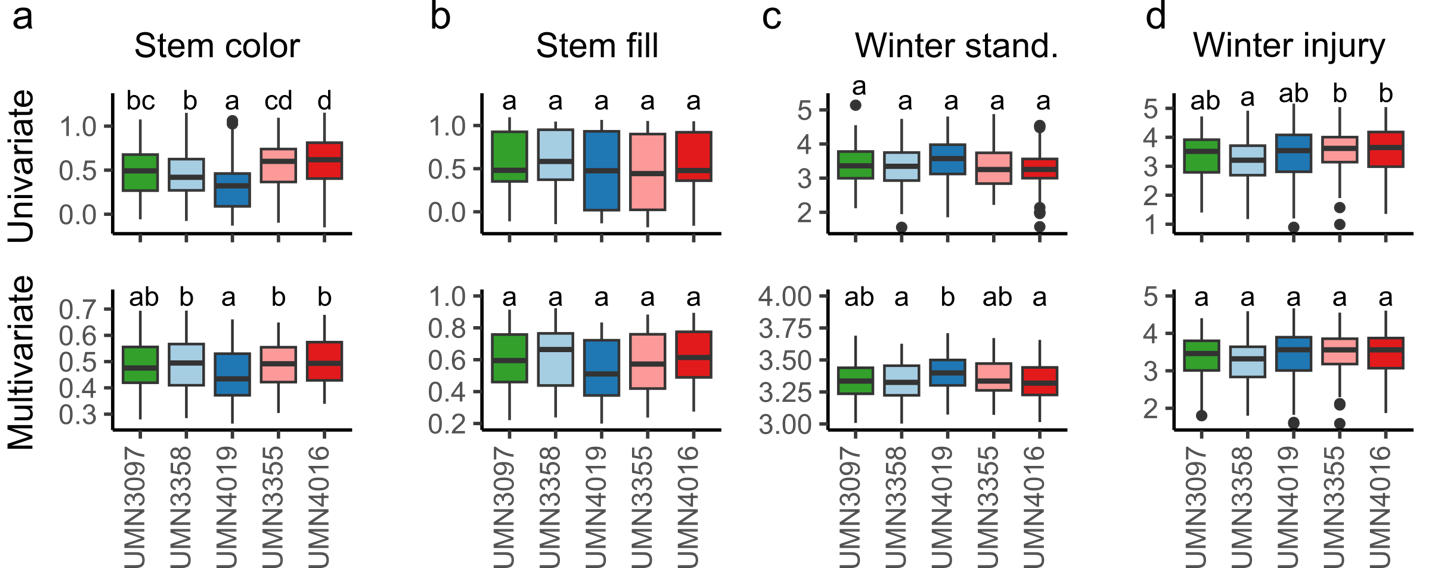


**Supplementary Figure 2. Boxplot of Best Linear Unbiased Estimates.** **a.** Stem color, **b.** Stem fill, **c.** Winter standability, **d.** Winter injury. Stem color was coded as 0 for a brown and 1 for a yellow stem. Stem fill was coded as 0 for a hollow and 1 for a solid stem. All traits were modeled as a continuous variable. Different letters stand for significantly different means (p-value < 0.05) using Tukey’s method for pairwise comparisons.


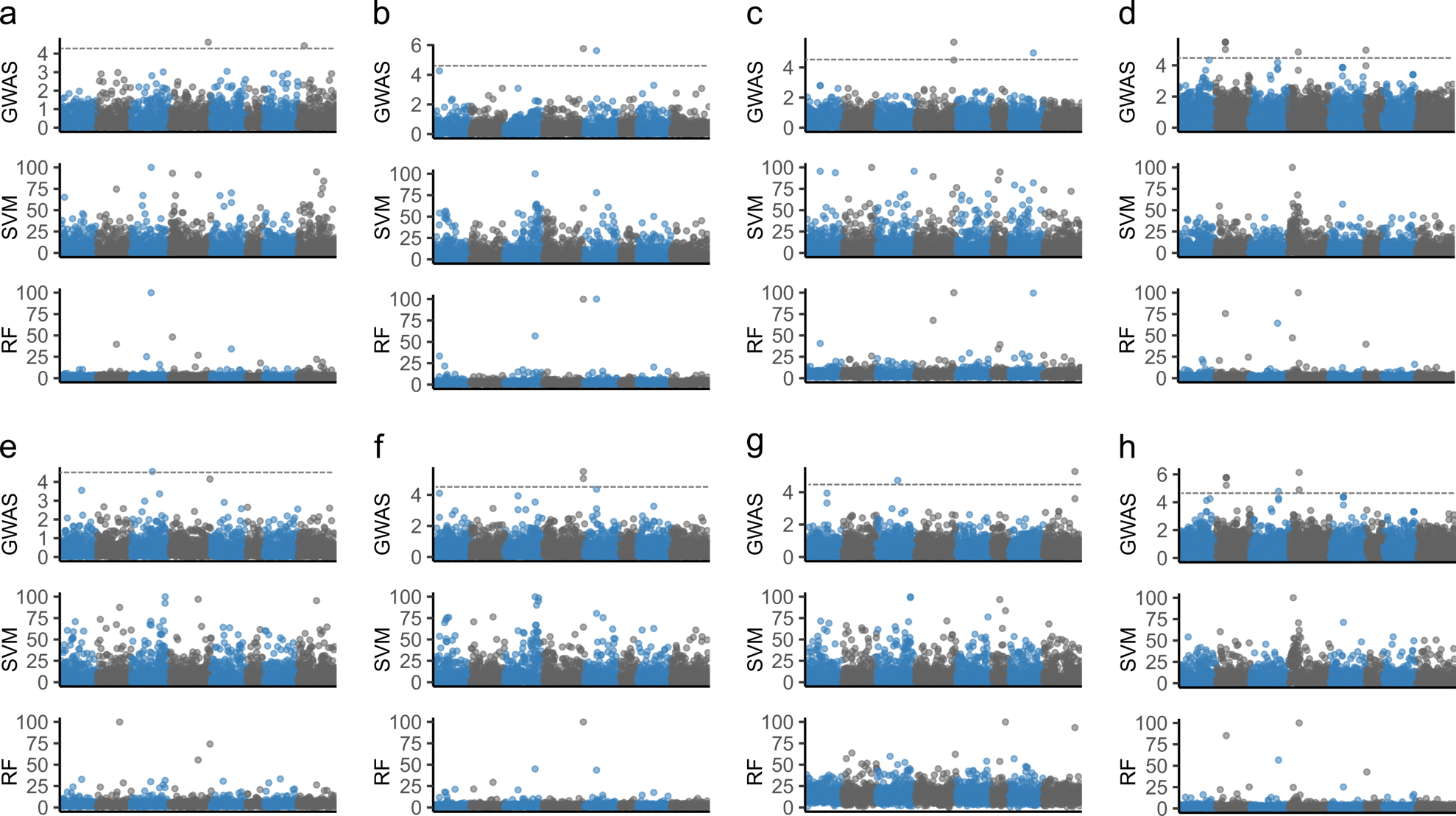


**Supplementary Figure 3. Manhattan plots for marker associations of four agronomic traits.** **a. and e.** Stem color, **b. and f.** Stem fill, **c. and g.** Winter standability and **d. and h.** Winter injury. Significant markers were identified using single trait (ST) (**a-d**) and multi trait (MT) (**e-h**) best linear unbiased estimators (BLUEs). GWAS scale correspond to -log_10_(p-values) and support vector machine (SVM) and random forest (RF) importance scores were scaled from 0 to 100.


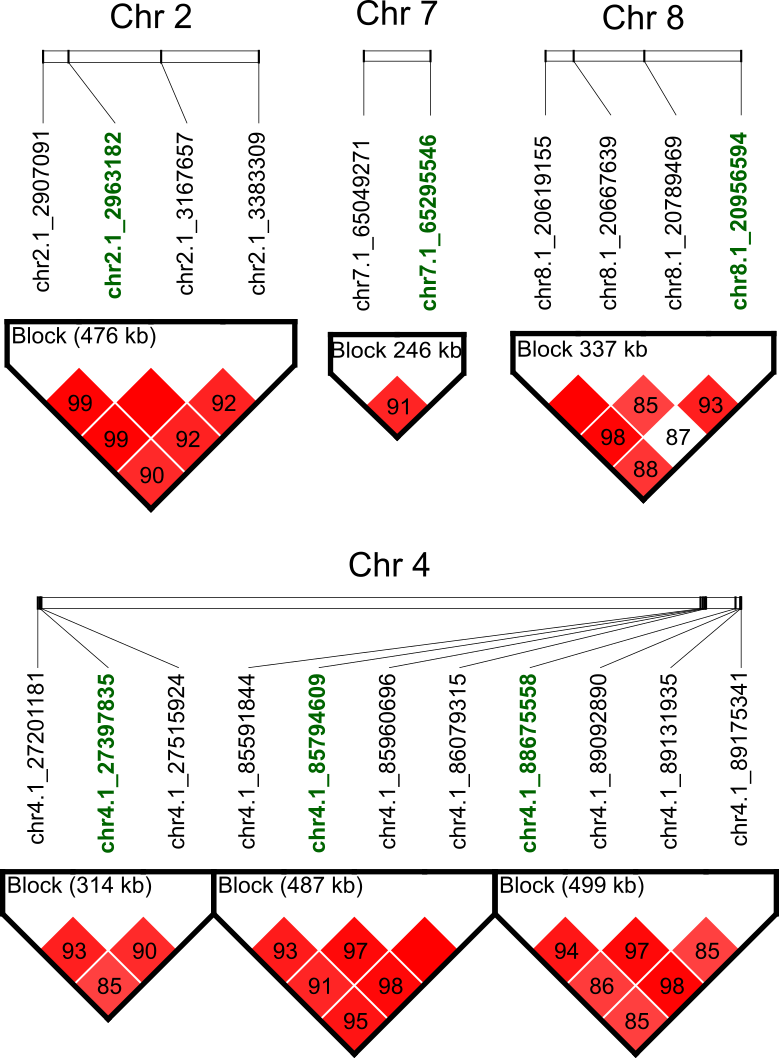


**Supplementary Figure 4. Linkage disequilibrium (LD) blocks in significant associated markers.** SNPs of interest (green color) were tested to identify LD blocks with Haploview v.4.2 (Barrett et al. 2005). Pairwise LD values (r^2^ × 100) were tested between SNPs of interest and their surrounding SNPs in a window size of 2 Mb. Numbers within the red squares represents the scores (D′) of the pairwise LD between SNPs, and an empty red squared indicates complete LD (100). Bright red indicates D′ = 1, LOD ≥ 2; white coloring indicates D’ < 1, LOD < 2.


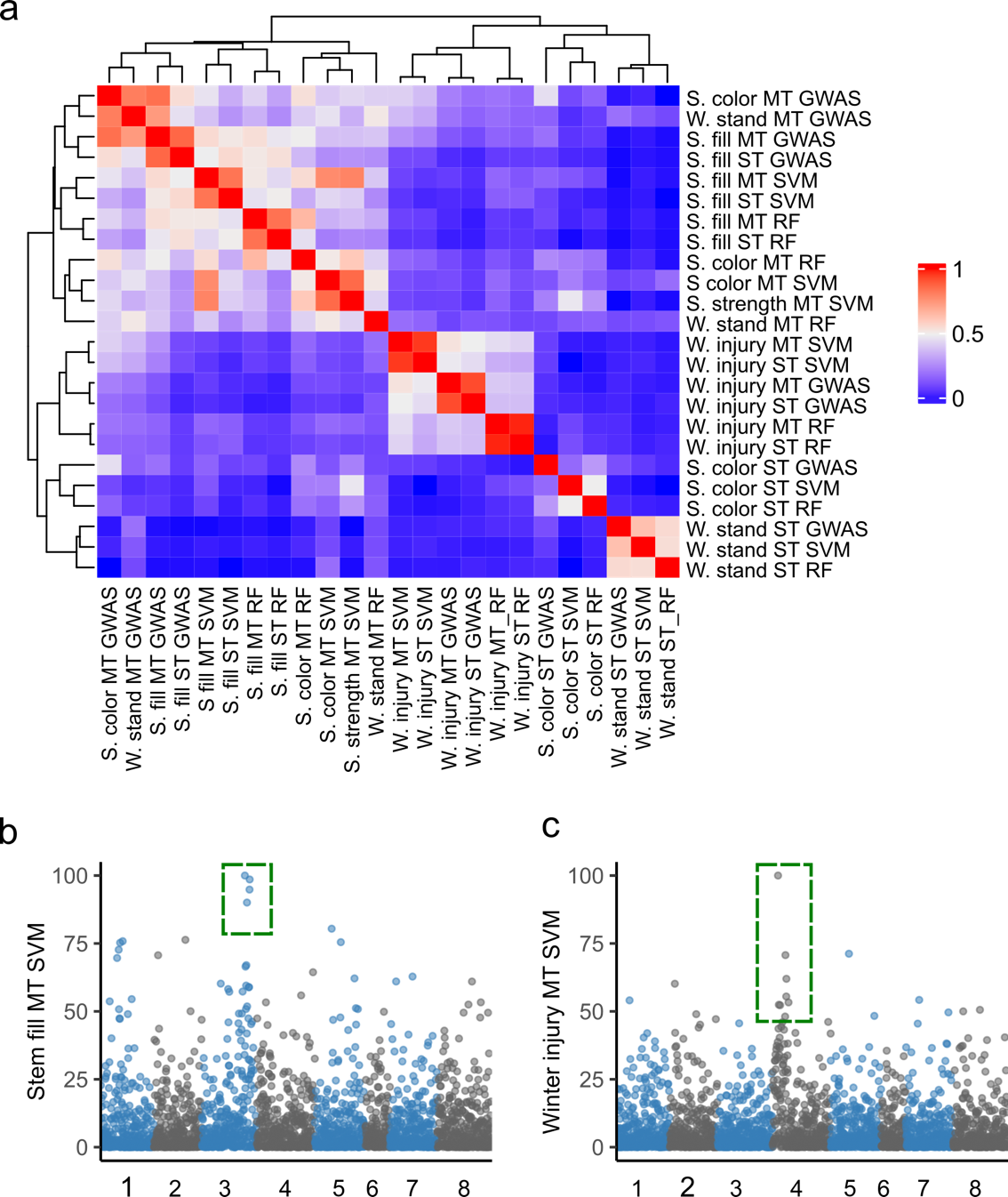


**Supplementary Figure 5. Correlation among variable importance scores from GWAS and machine learning models in different stem traits. a.** Pearson’s correlation of marker scores identified using single trait (ST) and multi trait (MT) best linear unbiased estimators (BLUEs) in different traits. GWAS scores correspond to -log10(p-values). Support vector machine (SVM) and random forest (RF) importance scores were scaled from 0 to 100. S. color, stem color; S. fill, stem color; W. stand, winter standability; W. injury, winter injury. **b.** Manhattan plot of SVM importance score in stem fill MT. Markers in green box of stem fill are chr3.1_78077889 = 100, chr3.1_84557406 = 98.6, chr3.1_84424131 = 94.8, chr3.1_80826259 = 90.1. **c.** Manhattan plot of SVM importance score in winter injury MT. Green boxes correspond to cluster of markers with high importance score in a close genomic region. Markers in green box of winter survival are chr4.1_17887905 = 100, chr4.1_26036161= 70.7, chr4.1_27397835 = 62, chr4.1_27201181 = 55.5, chr4.1_30353399 = 53.3, chr4.1_18963011 = 52.4, chr4.1_20508687 = 52.3.

# Supplementary Tables

**Supplementary Table 1.** Plant materials used to generate populations used in this study. six commercial alfalfa varieties used during the generation of Cycle 0 were 5312, Rushmore, Magnagraze, Wintergreen, Windstar, and WL 325HQ. H16 and H96 corresponds to high values of *in vitro* neutral detergent fiber digestibility (IVNDFD) at 16h and 96h respectively. L16 and L96 corresponds to low values of IVNDFD at 16h and 96h respectively. Geno correspond to the number of genotyped individuals by DArTag. Pheno correspond to the number of genotypes established on field for phenotyping.

| **Intermate** | | | **Cycle** | **Sel. Direction** | **Population** | **Geno** | **Pheno** |
| --- | --- | --- | --- | --- | --- | --- | --- |
| Six alfalfa varieties | | | 0 | Base population | UMN3097 | 298 | 100 |
| H16 | × | H96 | 1 | High Digestibility | UMN3355 | 363 | 100 |
| L16 | × | L96 | 1 | Low Digestibility | UMN3358 | 141 | 100 |
| H16 | × | H96 | 2 | High Digestibility | UMN4016 | 358 | 100 |
| L16 | × | L96 | 2 | Low Digestibility | UMN4019 | 342 | 100 |
| − |  | − | − | − | **Total** | 1,502 | 500 |

**Supplementary Table 2.** Wald test for fixed effects for four stem digestibility-associated traits. Terms were added sequentially. Winter stand, winter standability. Significant levels were coded as < 0.001 = ***, < 0.01 = **, < 0.5 = *, and > 0.5 = ns.

|  |  | Wald statistic or F-inc | | | |
| --- | --- | --- | --- | --- | --- |
| Source | df | Stem color | Stem fill | Winter stand | Winter injury |
| (Intercept) | 1 | 366.02*** | 696.68*** | 1183.01*** | 724.46*** |
| rep | 2 | 19.83*** | 3.25 ns | 7.42* | 18.27*** |
| gen | 499 | 647.84*** | 954.23*** | 476.44ns | 2299.70*** |

**Supplementary Table 3.** List of significant markers associated with the stem trait and candidate genes in a window of 84 kb. Bold rows correspond to gene coding regions located in markers same marker position. Distance corresponds to the distance of the gene coding region in kb downstream (-) or upstream (+) from the marker SNP. * Marker 4_85794609 was associated with stem color and winter standability.

| **Marker** | **Uniprot** | **Protein names** | **Distance** |
| --- | --- | --- | --- |
| *Stem color* |  |  |  |
|  | UPI000A2C1071 | Zinc finger BED domain-containing protein DAYSLEEPER-like | -44 |
|  | F6HXG1 | DYW domain-containing protein | -35 |
| **4_85794609*** | **UPI000DEDC8CC** | **Clu domain-containing protein** | **0** |
|  | A0A5E4EWL4 | VQ | +23 |
|  | UPI0012463AC8 | Glucuronoxylan 4-O-methyltransferase 1 | -23 |
|  | I1KC27 | Aminotransferase class V domain-containing protein | -19 |
| **8_20956594** | **UPI00078903CC** | **Replication factor C subunit 2** | **0** |
|  | A0A2I4DXU2 | IQ-domain 14 | +15 |
|  | UPI0010A55D37 | Phosphatidylinositol glycan anchor biosynthesis class U | +34 |
| *Stem fill* |  |  |  |
|  | A0A1S3UIS4 | Calnexin homolog isoform X2 | -34 |
|  | A0A4P1R310 | EF-hand domain-containing protein | -15 |
| 3_78077889 | UPI0001BE4214 | Succinate dehydrogenase assembly factor 2 | +4 |
|  | UPI000B3F15DD | Chromophore lyase CRL, chloroplastic | +13 |
|  | A0A2I4GPQ5 | Uncharacterized protein | +15 |
|  | A0A5B7B8X5 | CRAL-TRIO domain-containing protein | +25 |
|  | A0A2K3LDU0 | 40S ribosomal protein S12 | -15 |
|  | A0A2P5WBF6 | Poly(A)-specific ribonuclease (EC 3.1.13.4) | -9 |
| **4_88675558** | **A0A2I4GYU2** | **Factor of DNA methylation 1-like** | **0** |
|  | A0A151S6K4 | Aquaporin NIP6-1 | +22 |
|  | A0A444XBT8 | Golgi SNAP receptor complex member 1 | +29 |
|  | A0A445D677 | Isopentenyl phosphate kinase (EC 2.7.4.26) | -22 |
|  | UPI001263316A | Eukaryotic translation initiation factor 5A-2 | -15 |
|  | UPI000DEC16B7 | Mitotic checkpoint protein BUB3.3 isoform X3 | -7 |
| **5_32560792** | **UPI000DEC92ED** | **Cell division topological specificity factor** | **0** |
|  | UPI0002C2E47E | GDAP2 homolog isoform X1 | +31 |
| *Winter stand.* |  |  |  |
|  | A0A0B2PK56 | Sieve element occlusion B | -14 |
| **3_62442788** | **A0A445ABY6** | **Pre-rRNA-processing protein RIX1** | **0** |
|  | A0A5C7H1U4 | L-isoaspartate O-methyltransferase (EC 2.1.1.77) | +32 |
| **7_65295546** | **UPI00057A69DD** | **Sieve element occlusion B** | **0** |
|  | A0A444YT50 | AB hydrolase-1 domain-containing protein | +20 |
|  | A0A444YL65 | Uncharacterized protein | -37 |
| **8_74961533** | **UPI0010166183** | **RAVE complex protein Rav1** | **0** |
|  | A0A0S3RAP0 | Serpin domain-containing protein | +15 |
|  | A0A445BS04 | Serpin domain-containing protein | +23 |
|  | UPI0012632E13 | Kinesin light chain-related 1-like | +29 |
| *Winter injury* |  |  |  |
|  | UPI00103D80AA | Golgin subfamily A member 6-like protein 22 | -13 |
| **2_2963182** | **A0A0B2QM34** | **Pleiotropic drug resistance protein 1 (EC 3.6.3.-)** | **0** |
|  | G7IMF3 | Uncharacterized protein | +24 |
|  | UPI0003DE8008 | ATPase family AAA domain-containing protein 1-like | -41 |
|  | UPI0010AA2F3B | Metal-nicotianamine transporter YSL3-like | -36 |
|  | A0A0A0KN46 | Cytochrome b5 heme-binding domain-containing protein | -24 |
|  | UPI000809F86B | Rab GTPase-activating protein 22 isoform X3 | -18 |
| **3_74150027** | **A0A4D6NRG5** | **6-phosphogluconolactonase** | **0** |
|  | A0A2P5E6N3 | Ubiquitin | +13 |
|  | A0A1U8NA60 | Pyridoxal reductase, chloroplastic-like isoform X1 | +18 |
|  | UPI000C04CFA5 | Homeobox-leucine zipper protein | +25 |
|  | UPI0010A4324D | Transmembrane E3 ubiquitin-protein ligase FLY2-like | -48 |
|  | UPI0008FEB3A3 | Uncharacterized protein | -32 |
|  | UPI00092FC93A | Switch 2 | -25 |
|  | A0A2N9GIL2 | Complex 1 LYR protein | -10 |
| **4_27397835** | **UPI0010168B9C** | **O-acyltransferase WSD1-like isoform X2** | **0** |
| **6_11528532** | **UPI00051B12D6** | **3-ketoacyl-CoA synthase 20-like** | **0** |
